# Non-adherence to hemodialysis regimens among patients on maintenance hemodialysis in sub-Saharan Africa: an example from Cameroon

**DOI:** 10.1080/0886022X.2020.1826965

**Published:** 2020-10-07

**Authors:** Marie Patrice Halle, Musaga Nelson, Folefack Francois Kaze, Nda Mefo’o Jean Pierre, Tewafeu Denis, Hermine Fouda, Enow Gloria Ashuntantang

**Affiliations:** a Department of Internal Medicine, Faculty of Medicine and Pharmaceutical Science, Douala General Hospital, University of Douala, Douala, Cameroon; b Faculty of Health Sciences, University of Buea, Buea, Cameroon; c Faculty of Medicine and Biomedical Sciences, University of Yaoundé I, Yaoundé, Cameroon; d Faculty of Medicine and Pharmaceutical Science, University of Douala, Douala, Cameroon; e Department of Internal Medicine, Faculty of Medicine and Biomedical Sciences, Douala General Hospital Cameroon, University of Yaoundé I, Yaoundé, Cameroon; f Faculty of Medicine and Biomedical Sciences, Yaounde General Hospital, University of Yaounde I, Yaoundé, Cameroon

**Keywords:** Non-adherence, hemodialysis, regimens, sub-Saharan Africa, Cameroon

## Abstract

**Background:**

Non-adherence (NA) to hemodialysis regimens is one of the contributors to the high morbidity and mortality observed in patients with end-stage kidney disease (ESKD). We aimed to determine the prevalence and predictors of NA to hemodialysis (HD) regimens among patients on maintenance HD in Cameroon.

**Methods:**

A cross-sectional study in two HD centers in Cameroon was conducted from January to February 2016. Consenting patients on HD for ≥3 months were included. NA to fluid restriction was defined as a mean interdialytic weight gain (IDWG) in the past month >5.7% of the dry weight, NA to dietary restriction as a pre dialysis serum phosphorus >5.5 mg/dl in a patient on phosphate binders and who is well-nourished, and NA to HD sessions as skipping at least one session in the past month. The study was approved by the institutional ethics board.

**Results:**

A total of 170 (112 males) participants with a median age of 49 years (range 14–79) were included. The median dialysis vintage was 35 months (range 3–180 months). The prevalence of NA was 15.3% to fluid restriction, 26.9% to dietary restriction, and 21.2% to dialysis sessions. Age ≤49 years (*p* = .006, OR: 5.07, 95% CI: 1.59–16.20) and unmarried status (*p* = .041, OR: 2.63, 95% CI: 1.04–6.66) were independently associated with NA to fluid restrictions. No factor was associated with NA to dietary restrictions and HD sessions.

**Conclusions:**

NA to HD regimens is common amongst patients in Cameroon. Younger age and being unmarried were the predictors of NA to fluid restriction.

## Introduction

Chronic kidney disease (CKD) is a public health problem worldwide with approximately 13% of adults affected [1]. CKD progress in five stages and at end stage renal replacement therapy (RRT) through either dialysis or renal transplantation is necessary for survival [2]. RRT is a high-costly treatment and hemodialysis (HD) is the most commonly used modality in the world [3]. Adequate HD improves the quality of life of patients and the success of the therapy needs the patient’s cooperation and depends on their adherence to medication, to diet, to dialysis sessions, and to fluid restrictions [4,5]. Non-adherence (NA) to these regimens is a frequent phenomenon and reported prevalence varies depending on the continent and the parameter studied, and it is an important cause of morbidity, and mortality amongst patients on maintenance HD [5–16].

Excessive fluid intake leads to hypervolemia which can result in high blood pressure and pulmonary edema, increasing cardiovascular damage, and death [8,17,18]. Several socio demographic, psychological, and clinical factors are associated with NA to HD regimens [9,19–21]. The reported prevalence rates of NA to fluid restriction varied from 7.4% to 75.3% worldwide depending on the definition used [6,9,11,12].

In sub-Saharan Africa, lack of funding and poverty is a major barrier to achieve adequate dialysis [21,22]. HD is the only modality of RRT available in Cameroon with 12 centers in 2015 and is partly subsidized since 2002 [23]. Despite the state subsidies, management of end-stage kidney disease (ESKD) in Cameroon is challenging. Patients have no medical insurance and the out of pocket payment for medications, routine laboratory test, and hospitalizations are very high and not affordable for the majority [24]. Consequently, morbidity of these patients is high. Kaze et al. found that hypertensive crisis (14%), muscle cramps (22%) were frequent acute HD complications [25]. Heart failure was associated with high interdialytic weight gain (IDWG) [26] and hyperkalemia was frequent in HD patients before the second HD session [27]. Also, mortality of patients on maintenance HD in Cameroon is high ranging from 26.6% to 57.58% with some of the principal causes being uremia and catheter-related sepsis [28,29]. Despite the importance of adherence to dialysis regimen, data are inexistent in our setting. Therefore, we aimed to determine the prevalence and associated factors to NA to dialysis regimens in Cameroon.

## Methods

### Study setting and participants

This was a hospital-based cross sectional and analytical study carried out from 1 January to 29 February 2016 in the HD centers of the Buea Regional Hospital (BRH) and Douala General Hospital (DGH) in Cameroon, a secondary and tertiary hospital, respectively. The two hospitals are each run by at least a nephrologist, general practitioners, and nurses. A general dietician is available in the DGH but not in BRH. Approximately 99% of patients in both centers undergo two dialysis sessions of 4 h each per week while a few received three sessions per week. Both centers use Fresenius^®^ generators 4008 S (Fresenius Medical Care, Hamburg, Germany). Patients are weighed before and after each HD session and each patient’s HD chart is used for follow-up. A consecutive sample of consenting patients with ESKD and undergoing maintenance HD for at least 3 months were included in the study. Patients with acute illnesses were excluded from the study. The study was approved by decision no. IEC-UD/486/02/2016/T of the Institutional Ethics Committee for Research on Human Health of the University of Douala.

### Data collection

All ESKD patients on maintenance HD in the two centers were approached during their dialysis sessions and their consent sought. Consenting patients who were on HD for at least 3 months and more were enrolled in the study. Each participant was interviewed and their medical records reviewed to obtain relevant socio-demographic data (age, gender, educational level, income level, marital status, living status, and residence) and clinical data (use of phosphate binders and potassium binders, prior information on fluid and dietary restrictions, residual diuresis, dietician consultations, and skipped. Other clinical variables collected included comorbidities, etiology of ESKD, HD prescription, date of initiation of HD, vascular access, and dry weight) which were filled on the data collection tool. The mean IDWGs were calculated for a period of 1 month prior to the date of questionnaire administration for each patient. This was done using the pre- and post-dialysis weights recorded in patients’ dialysis monitoring charts. The mean interdialytic weight was then expressed as a percentage of the dry weight noted in the patient’s dialysis monitoring charts. From each participant, 4 ml of venous blood samples were collected at the beginning of the second session of the week for the dosage of serum phosphorus in the biochemistry laboratory of the DGH using the Cobas C311 Roche Hitachi.

### Definition of operational terms

Patients with low serum albumin levels (less than 35 g/l) and low serum phosphorus(less than 2.5 mg/dl) levels were considered as being malnourished. NA to fluid restriction was defined as a mean IDWG in the past month >5.7% of the dry weight. NA to dietary restriction was considered in a patient on phosphate binders with pre-dialysis serum phosphorus > 5.5 mg/dl in a patient on phosphate binders in the absence of malnutrition. NA to HD sessions was defined as skipping at least one HD session in the past month.

### Statistical analysis

Data were analyzed with the aid of IBM statistical package for the social sciences (IBM SPSS, Armonk, NY) software version 20.0. Categorical variables were expressed as frequencies and percentages while the continuous variables were described using mean ± SD, median ± 25th–75th, QR and range. The prevalence of NA to the different HD regimens was calculated by dividing the number of non-adherent participants by the total number of participants. Bivariate and multivariate logistic regression was used to identify the predictors of NA to different HD regimens. The level of statistical significance was set at *p* value ˂.05 with 95% confidence interval.

## Results

### General characteristics of participants

A total 233 out of 240 patients were eligible for the study in both centers amongst which 179 initially consented to participate in the study, 9 were excluded, and 170 participants were included in the final analysis. The median age of participants was 49 years (14 − 79 years), 112/170 (65.9%) were males, 130/170 (76.5%) were married, 66/170 (38.8%) had at least a secondary education, and 87/170 (51.2%) were employed (Table 1). A total of 150/170 (90%) resided in the town of dialysis, 52/170 (30.6%) had a monthly income less than 50,000 FCFA and only one (0.6%) patient had a health insurance. The median duration on dialysis was 35 months (range 3–180 months), 90/170(52%) of patients had a residual diuresis. Hypertension 33/169 (19.5%) and muscle cramps 32/169 (18.9%) were the most common predialytic complications and 105/170 (61.8%) were on phosphate binders.

**Table 1.** Socio demographic characteristics of the study participants (*N* = 170).

Variable	*N*	%
Age		
Median (years)	49	
Gender		
Male	112	65.9
Female	58	34.1
Marital status	N	
Married	130	76.5
Unmarried	40	23.5
Level of education		
None	6	3.5
Primary	32	18.8
Secondary	66	38.8
High school	20	11.8
University	46	27.1
Employment status		
Employed	87	51.2
Unemployed	50	29.4
Retired	33	19.4
Monthly income (FCFA)		
No income	36	21.2
<50,000	52	30.6
50,000–100,000	42	24.7
100,000–150,000	24	14.1
>150,000	16	9.4
Source of funding		
Self	66	38.8
Insurance	1	0.6
Family member	56	32.9
Self and family member	47	27.6
Living status		
Family	167	98.1
Friends	1	0.6
Alone	2	1.2

A total of 163/170 (95.9%) participants had received information on fluid and dietary restriction mostly from the nephrologist (75%). However, 152/170 (89.4%) patients had good knowledge on fluid restriction, 110/170 (64.7%) of patients had bad knowledge of dietary restrictions, and 55/170 (32.4%) had been consulted by a dietician (Table 2).

**Table 2.** Clinical characteristics of the study population.

Variable	*N*	%
Vascular access		
AV fistula	153	90
TCVC	13	7.6
PCVC	3	1.8
AV graft	1	0.6
Sessions per week		
2	168	98.8
3	2	1.2
Residual diuresis		
Yes	90	52.9
Use of phosphate binders		
Yes	105	61.8
Knowledge on fluid restriction		
Good	152	89.4
Average	4	2.4
Insufficient	6	3.5
Bad	8	4.7
Knowledge on dietary restriction		
Good	13	7.6
Average	27	15.9
insufficient	20	11.8
Bad	110	64.7

TCVC: temporary central venous catheter; PCVC: permanent central venous catheter.

### Prevalence and predictors of NA to hemodialysis regimens in the study population

The mean IDWG was 2.89 ± 0.87 kg. Prevalence of NA to fluid restriction was 15.3% (26/170). The median serum phosphorus levels were 4.0 mg/dl (range 0.9– 9.2). Of the 105 patients on phosphate binders, 42 were malnourished thus excluded from the prevalence analysis. Of the 63 patients left, 17 were non-adherent giving a prevalence of 26.9%. A total of 36 participants had skipped ≥ 1 session in the month prior to the date of enrollment. The number of times participants had skipped sessions ranged from 1 to 5 with a mean of 2.14 times. The prevalence of NA to HD sessions was therefore 21.2% (36/170). On bivariate analysis, absence of residual diuresis was significantly associated (*p* value = .012, OR = 5.06 95% CI = 1.42–17.96) with NA to dietary restriction with a borderline *p* (0.05) on multivariate analysis (Table 3).

**Table 3.** Prevalence of non-adherence to hemodialysis regimens.

Variable	Adherence *n* (%)	Non-adherence *N* (%)
Fluid restriction (=170)	144 (84.7%)	26 (15.3%)
Dietary restriction (*n* = 63)	46 (73.1%)	17 (26.9%)
Hemodialysis session (*n* = 170)	134 (78.8%)	36 (21.2%)

Age ≤49 years (*p* value = .006, aOR = 5.07, 95% CI = 1.59–16.20) and being unmarried (*p* value = .04, aOR  =  2.63, 95% CI = 1.04–6.66) were both associated to NA to HD regimens on bivariate and multivariate analysis (Table 4). None of the variables was associated with NA to HD sessions (Table 4 and Table 5).

**Table 4.** Predictors of non-adherence to dietary restriction on multivariate analysis (*n* = 63).

Variable	Participants NA to dietary restriction	Multivariate analysis	
		aOR (95% CI)	*p* Value
Duration on hemodialysis /months			
>35	5	Ref	
≤35	12	0.566 (0.148–2.159)	.405
Residual diuresis			
Yes	4	Ref	
No	13	3.965 (0.998–15.745)	.050

aOR: adjusted odds ratio; CI: confidence interval.

**Table 5.** Predictors of non-adherence to fluid restriction and to sessions on multivariate logistic regression (*N* = 170).

Multivariate analysis						
	Participants non-adherent		Fluid		Session	
Variable	Fluid	Sessions	aOR (95% CI)	*p* Value	aOR (95% CI)	*p* Value
Age						
>49 years	4	12	Ref		Ref	
≤49 years	22	24	5.07 (1.59 − 16.20)	.006	1.905 (0.828 − 4.38)	.13
Income						
>100,000	8	12			Ref	
≤100,000	18	24	–	–	0.503 (0.217–1.166)	.109
Marital status						
Married	13	14	Ref		Ref	
Unmarried	13	22	2.63 (1.04–6.66)	.04	2.19 (0.928–5.151)	.074
Duration on hemodialysis /months						
>35	11	15			Ref	
≤35	15	21	–	–	1.675 (0.775–3.618)	.189

aOR: adjusted odds ratio; CI: confidence interval.

## Discussion

The aim of this study was to determine the prevalence and predictors of NA to HD regimens in Cameroon. We found a prevalence rate of 15.3% for NA to fluid restriction, 26.9% for dietary restriction, and 21.2% for HD sessions. Young age and unmarried status were independent predictors of NA to fluid restriction. No factor was associated with NA to dietary restriction and HD sessions.

The reported prevalence rates of NA to fluid restriction varied from 7.4% to 75.3% worldwide depending on the definition used [6,9,11,12]. The highest rate has been reported when subjective methods of assessment of NA were used [6]. We found a prevalence rate of 15.3% which is within the reported range of most studies that used the same definition as in the present study [9,30–34]. The subjective assessment tool which is a self-administered questionnaire entails computing the number times within a period of 14 d when patient disrespected the restriction [35]. This may, therefore, overestimate. Our prevalence rate is however much lower compared to similar facilities where patients undergo two dialysis sessions a week [7]. Fewer dialysis sessions a week are associated with long interdialytic periods and thus the likelihood of having high IDWGs. Our findings may reflect the good knowledge (89.4%) of participants in this study about fluid restriction observed. However, Safdar et al. in Pakistan reported a high prevalence (64%) of NA to fluid restriction among patients undergoing two weekly HD sessions despite good knowledge [36]. While other factors may influence behavior, the higher prevalence rates reported by Safdar et al. may also be due to a difference in study design. They evaluated only four of eight interdialytic weights of the month and also defined weight gain as an absolute change in weight rather than a percentage of dry weight. Prevalence rates lower than 10% have been reported elsewhere especially in facilities which practice three dialysis sessions weekly, and have renal dieticians, health educators, psychologists, and a high physician to patient ratio allowing for better care [9,12,34]. The absence of renal dieticians and psychologists in the study setting as well as all HD units in Cameroon renders continuous counseling difficult.

Similarly, the prevalence rate of NA to dietary restriction in previous studies range from 5.5% to 81.4% with the highest rates obtained when subjective methods are used [6]. We found a prevalence of 26.9% for NA to dietary restriction which is in consonance with rates reported for most studies which used serum phosphorus as a surrogate for NA to dietary restriction [12]. Studies which have used serum potassium alone or combined with serum phosphorus have found rates ranging from 5.5% to 52% [9,12]. Lower prevalence rates have been observed especially in centers which practice three HD sessions weekly. A similar situation prevails in centers which have renal dieticians, health educators, psychologists, and a high physician to patient ratio allowing for better care [30,31,34,37]. Thrice weekly dialysis sessions result in better clearance of serum phosphorus and potassium compared to twice weekly dialysis sessions. Our findings may overestimate or underestimate the prevalence of NA to dietary measures since we used serum phosphorus as a surrogate. Phosphorus-rich foods are also protein-rich foods, such that overzealous adherence to dietary restriction of phosphorus-rich foods may cause protein-energy malnutrition and low phosphorus levels [38]. Excluding malnourished patients with low serum phosphorus levels from the analysis may miss some patients who were adherent. Dietary restriction without phosphate binders is usually not enough to control hyperphosphoremia without running the risk of malnutrition in patients on intermittent HD.

The reported prevalence rates of NA to HD sessions vary from 0.6% when defined as shortening a session for more than 10 min [9] to as high as 55.9% when defined skipping one or more sessions in the past month [33]. We found a prevalence of 21.2% for NA to dialysis sessions which are within the reported range. Lower prevalence has been reported in studies done in the western world [9,31,35,37]. The less than 1% health insurance coverage for our patients and financial constraints may explain these rates. Despite government subsidies for HD session in Cameroon, the out pocket expenditure remain high as reported by Halle et al. [24] and may therefore constitute barriers to accessing treatment. In this study, over 50% of participants had a monthly income less than 50,000 FCFA and about 60% were funded by family and friends renders health education for behavioral change difficult.

Adherence to HD regimens is multifactorial including age, sex, duration on dialysis, economic and psychosocial factors. NA to fluid restriction in our study was common in younger and unmarried patients just like other studies have linked NA to age and marital status [6,12,31,34,37,39–44]. Middle-aged and younger patients may have other priorities in life. Due to work and other social commitments may find compliance to chronic therapies difficult. Low adherence has also been reported in adolescents with various chronic conditions usually linked to lack of understanding or related to their parents and guardians [9]. The desire to live normal lives like their peers may account for these findings [45]. In consonance with previous reports, we found unmarried patients likely to be non-adherent to fluid restriction [34]. It is plausible that being married provides social support and possible reminders to foster compliance. Contrary to other reports, gender, employment status, knowledge, and duration on dialysis was not associated with NA in this study [36,46,47].

We did not find an association between the socio-demographic and clinical variables and NA to dietary restrictions and HD sessions. This is contrary to findings obtained in other studies where younger age, lower levels of education, shorter dialysis vintage, employed status, being unmarried, and living alone have been associated with NA to dietary restrictions and HD sessions [9,30,34,37].

## Conclusion

NA to HD regimens is common amongst patients on maintenance HD in Cameroon. Younger age and being unmarried were the predictors of NA to fluid restriction. No factor was associated with NA to dietary restriction and HD sessions in this study.
